# SNP interactions of *Helicobacter pylori*-related host genes *PGC, PTPN11, IL1B*, and *TLR4* in susceptibility to gastric carcinogenesis

**DOI:** 10.18632/oncotarget.4231

**Published:** 2015-05-22

**Authors:** Caiyun He, Huakang Tu, Liping Sun, Qian Xu, Yuehua Gong, Jingjing Jing, Nannan Dong, Yuan Yuan

**Affiliations:** ^1^ Tumor Etiology and Screening Department of Cancer Institute and General Surgery, the First Affiliated Hospital of China Medical University, and Key Laboratory of Cancer Etiology and Prevention (China Medical University), Liaoning Provincial Education Department, Shenyang, China; ^2^ Department of Molecular Diagnostics, Sun Yat-Sen University Cancer Center, State Key Laboratory of Oncology in South China, Collaborative Innovation Center for Cancer Medicine, Guangzhou, China; ^3^ Department of Epidemiology, Rollins School of Public Health, Emory University, Atlanta, GA, USA

**Keywords:** Helicobacter pylori, gastric cancer, atrophic gastritis, susceptibility, interaction

## Abstract

A series of host genes that respond to *Helicobacter pylori* (*H. pylori*) infection are involved in the process of gastric carcinogenesis. This study sought to examine interactions among polymorphisms of *H. pylori-*related genes *PGC, PTPN11, TLR4*, and *IL1B* and assess whether their interaction effects were modified by *H. pylori* infection. Thirteen polymorphisms of the aforementioned genes were genotyped by the Sequenom MassARRAY platform in 714 gastric cancer patients, 907 atrophic gastritis cases and 1276 healthy control subjects. When we considered the host genetic effects alone, gene–gene interactions consistently decreased the risks of gastric cancer and/or atrophic gastritis, including three two-way interactions: *PGC* rs6912200-*PTPN11* rs12229892, *PGC* rs4711690-*IL1B* rs1143623 and *PTPN11* rs12229892-*IL1B* rs1143623 and a three-way interaction: *PGC* rs4711690-*PGC* rs6912200-*PTPN11* rs12229892. When the effect modification of *H. pylori* infection was evaluated, the cumulative effects of the aforementioned three-way interaction on atrophic gastritis susceptibility switched from being beneficial to being risky by the status of *H. pylori* infection. These data showed that SNP interactions among *H. pylori*-related genes *PGC, PTPN11*, and *IL1B*, are associated with susceptibility to gastric carcinogenesis. Moreover, we provided important hints of an effect modification by *H. pylori* infection on the cumulative effect of *PGC* and *PTPN11* polymorphisms. Functional experiments and further independent large-scale studies especially in other ethnic populations are still needed to confirm our results.

## INTRODUCTION

The genetic basis of susceptibility to gastric cancer is the cumulative result of germ-line variations at many different loci, with each gene only having a small effect [1]. In typical case-control association studies of gastric cancer, candidate genes are examined either by evaluating one marker at a time or by forming haplotypes over multiple neighboring loci in and around one gene [2]. There are limited data regarding the influence of gene–gene interactions on gastric cancer risk. One important type of gene–gene interaction is epistasis, in which the genes interact with one another and modify each other's behavior [3]. In fact, understanding epistatic interactions may be the key to understanding complex diseases. Knowledge of gene–gene interactions could reveal substantial hidden heritability within the architecture of gastric cancer susceptibility [4].

*Helicobacter pylori* is a confirmed environmental risk factor for gastric cancer and its precursors, such as atrophic gastritis and dysplasia [5]. It exhibits carcinogenic effects on gastric epithelial cells and these effects are mediated by a series of virulence factors and toxic components [6]. The host response to these bacterial components differs between individuals and is extremely important for determining gastric cancer predisposition [6]. It is becoming increasingly clear that several specific host genes, such as *PGC* (pepsinogen C), *PTPN11* (protein tyrosine phosphatase, non-receptor type 11), *TLR4* (Toll-like receptor 4), and *IL1B* (interleukin-1B), are involved in the response to *H. pylori* infection and are currently identified as susceptibility genes for gastric cancer [6, 7].

PTPN11 and TLR4 are crucial components of the gastric epithelial cell signaling pathway and respond to the virulence factors LPS (lipopolysaccharide) and CagA (cytotoxin-associated antigen) of *H. pylori,* respectively [8]. Such host-microbe interactions can activate the NF-kB (nuclear factor-kappa B) and MAPK (mitogen-activated protein kinase) signaling pathways, which promote the production of the proinflammatory factor IL-1β or induce aberrant apoptosis or proliferation [8-11]. PGC, a well-known biomarker for the differentiation of gastric epithelium cells, has recently been recognized as a surrogate for *H. pylori* infection in the stomach [12-14]. The aforementioned host genes appear to have pleiotropic effects on the signal transduction of inflammatory or immune reactions, proliferation, apoptosis, and cell differentiation [8, 14-16].

Individual genetic effects of 13 single nucleotide polymorphisms (SNPs) in *PGC*, *PTPN11*, *TLR4*, and *IL1B* on the susceptibility to gastric cancer and atrophic gastritis have been reported in our previous studies [7, 17]. In this study, we investigated the potential gene–gene interactions among those SNPs and assessed whether the effects of these interactions were modified by *H. pylori* infection. To our knowledge, this is the first study to investigate interactions between *H. pylori*-related genes as risk factors for gastric carcinogenesis.

## RESULTS

### Main effects of single polymorphisms of PGC, PTPN11, TLR4, and IL1B

In our previous studies [7, 17], we found that the genotype frequencies of five tagSNPs of *PGC* (rs4711690, rs6458238, rs9471643, rs3789210, and rs6939861) in gastric cancer and/or atrophic gastritis were significantly different from those in controls. Moreover, *H. pylori* infection status affected the ORs of three tagSNPs (*PGC* rs4711690, *PGC* rs6912200, and *PTPN11* rs12229892) for the development of gastric cancer or atrophic gastritis. There was no overall genetic effect on risk for *PGC* rs6912200 and rs6941539; *PTPN11* rs12229892; *IL1B* rs1143623, rs1143627 and rs1143643; or *TLR4* rs11536878 and rs10983755 in our study population.

### Two-way interactions between polymorphisms of *PGC, PTPN11, TLR4,* and *IL1B*

Using a combined genotype comprising the most common SNP for each gene, two-way gene–gene interactions among the 13 tagSNPs of our genes of interest were assessed. In the two-way interaction analyses involving *PGC* (Table 1 and Supplementary Tables 2 and 3), the most significant interaction was between *PGC* rs6912200 and *PTPN11* rs12229892. This interaction was associated with altered risks for the development of both gastric cancer and atrophic gastritis (gastric cancer risk: *P* value for interaction = 0.017, interaction index = 1.96; atrophic gastritis risk: *P* value for interaction = 0.010, interaction index = 1.97). In addition, *PGC* rs4711690 showed a significant interaction with *IL1B* rs1143623 in relation to gastric cancer risk (*P* value for interaction = 0.047, interaction index = 0.65). In the two-way analyses involving *PTPN11* (Table 2), *PTPN11* rs12229892 showed a significant interaction with *IL1B* rs1143623, influencing gastric cancer risk (*P* value for interaction = 0.034, interaction index = 1.64). In the two-way analyses between *TLR4* and *IL-1B*, no statistically significant interaction was observed (Supplementary Table 4).

**Table 1:** Two-way interaction effect between *PGC* tagSNPs and *PTPN11* and *IL1B* tagSNPs on the risks of gastric cancer and atrophic gastritis

*PGC* tagSNP	For GA vs. CON					For GC vs. CON				
		*PTPN11* rs12229892		*IL1B* rs1143623		*PTPN11* rs12229892		*IL1B* rs1143623	
		GG	GA/AA	GG	GA/AA	GG	GA/AA	GG	GA/AA
rs4711690									
CC	Controls/Cases	227/129	476/290	252/143	452/277	227/180	476/359	252/183	452/357
	OR(95%CI)	1(ref)	1.21(0.90,1.61)	1(ref)	1.18(0.89,1.56)	1(ref)	0.89(0.69,1.15)	1(ref)	1.05(0.81,1.35)
CG/GG	Controls/Cases	164/93	393/196	198/117	362/171	164/103	393/258	198/129	362/232
	OR(95%CI)	1.08(0.75,1.57)	0.88(0.65,1.20)	1.08(0.77,1.52)	0.82(0.61,1.12)	0.70(0.50,0.98)	0.73(0.56,0.96)	0.80(0.59,1.10)	0.80(0.61,1.05)
		P for interaction=0.089, interaction index=0.68		**P for interaction=0.047, interaction index =0.65**		P for interaction=0.420, interaction index=1.18		P for interaction=0.797, interaction index =0.95	
rs6458238									
GG	Controls/Cases	325/183	710/402	361/215	678/369	325/246	710/526	361/264	678/509
	OR(95%CI)	1(ref)	1.04(0.82,1.33)	1(ref)	0.93(0.74,1.18)	1(ref)	0.92(0.74,1.14)	1(ref)	0.98(0.80,1.21)
AG/AA	Controls/Cases	67/39	161/87	90/47	138/79	67/39	161/92	90/49	138/83
	OR(95%CI)	0.96(0.60,1.55)	0.95(0.67,1.35)	0.80(0.52,1.22)	0.96(0.67,1.37)	0.65(0.41,1.04)	0.70(0.51,0.97)	0.64(0.42,0.96)	0.78(0.56,1.10)
		P for interaction=0.860, interaction index=0.95		P for interaction=0.355, interaction index=1.29		P for interaction=0.573, interaction index=1.17		P for interaction=0.405, interaction index=1.25	
rs9471643									
GG/CC	Controls/Cases	234/141	536/316	272/164	498/293	234/185	536/385	272/201	498/367
	OR(95%CI)	1(ref)	1.07(0.81,1.41)	1(ref)	1.09(0.84,1.43)	1(ref)	0.87(0.70,1.12)	1(ref)	1.01(0.80,1.29)
GC	Controls/Cases	157/80	332/173	176/96	315/155	157/99	332/231	176/109	315/223
	OR(95%CI)	0.86(0.59,1.25)	0.82(0.60,1.12)	0.95(0.67,1.35)	0.77(0.57,1.05)	0.78(0.54,1.07)	0.80(0.60,1.05)	0.84(0.61,1.16)	0.90(0.69,1.17)
		P for interaction=0.639, interaction index=0.90		P for interaction=0.183, interaction index=0.74		P for interaction=0.395, interaction index=1.19		P for interaction=0.801, interaction index=1.05	
rs3789210									
CC	Controls/Cases	199/162	470/323	241/183	429/302	199/201	407/408	241/203	429/408
	OR(95%CI)	1(ref)	0.88(0.67,1.15)	1(ref)	0.93(0.71,1.20)	1(ref)	0.77(0.60,0.99)	1(ref)	1.06(0.93,1.36)
GC/GG	Controls/Cases	76/44	144/111	79/50	141/104	76/47	144/115	79/67	141/95
	OR(95%CI)	0.73(0.46,1.17)	0.95(0.67,1.350	0.77(0.50,1.19)	0.99(0.71,1.40)	0.58(0.38,0.91)	0.69(0.49,0.96)	0.92(0.62,1.36)	0.76(0.54,1.06)
		P for interaction=0.162, interaction index=1.48		P for interaction=0.230, interaction index=1.39		P for interaction=0.110, interaction index=1.54		P for interaction=0.317, interaction index=0.77	
rs6912200									
CC	Controls/Cases	55/53	172/113	77/66	148/98	55/71	172/127	77/72	148/126
	OR(95%CI)	1(ref)	0.56(0.35,0.91)	1(ref)	0.71(0.45,1.11)	1(ref)	0.51(0.32,0.79)	1(ref)	0.85(0.56,1.31)
CT/TT	Controls/Cases	217/152	442/319	243/164	419/308	217/176	442/393	243/196	419/374
	OR(95%CI)	0.57(0.36,0.91)	0.60(0.41,0.98)	0.68(0.45,1.03)	0.79(0.53,1.16)	0.66(0.43,1.01)	0.66(0.44,0.98)	0.90(0.60,1.33)	0.97(0.67,1.41)
		**P for interaction=0.017, interaction index=1.96**		P for interaction=0.063, interaction index=1.64		**P for interaction=0.010, interaction index=1.97**		P for interaction=0.341, interaction index=1.27	
rs6939861									
GG	Controls/Cases	117/87	272/159	146/92	242/153	117/98	272/197	146/108	242/190
	OR(95%CI)	1(ref)	0.76(0.53,1.10)	1(ref)	0.98(0.69,1.40)	1(ref)	0.78(0.55,1.10)	1(ref)	1.00(0.72,1.40)
AG/AA	Controls/Cases	136/112	321/261	163/131	295/242	136/142	321/310	163/155	295/295
	OR(95%CI)	1.06(0.71,1.58)	1.12(0.79,1.58)	1.26(0.87,1.82)	1.33(0.96,1.86)	1.25(0.85,1.82)	1.07(0.77,1.49)	1.27(0.90,1.81)	1.32(0.96,1.81)
		P for interaction=0.186, interaction index=1.39		P for interaction=0.728, interaction index=1.09		P for interaction=0.687, interaction index=1.10		P for interaction=0.886, interaction index=1.03	
rs6941539									
CC	Controls/Cases	203/143	434/316	236/166	402/293	203/169	434/359	236/190	402/339
	OR(95%CI)	1(ref)	1.04(0.78,1.37)	1(ref)	1.08(0.82,1.41)	1(ref)	0.89(0.69,1.16)	1(ref)	0.99(0.77,1.28)
CT/TT	Controls/Cases	70/63	176/117	82/67	164/112	70/77	176/161	82/79	164/159
	OR(95%CI)	1.28(0.83,1.97)	0.98(0.70,1.38)	1.23(0.82,1.86)	1.03(0.74,1.44)	1.30(0.87,1.96)	0.98(0.72,1.34)	1.14(0.77,1.67)	1.17(0.86,1.48)
		P for interaction=0.275, interaction index=0.75		P for interaction=0.335, interaction index=0.78		P for interaction=0.494, interaction index=0.84		P for interaction=0.882, interaction index=1.04	

All tests were adjusted by age, sex and *H. pylori* infection. Statistically significant interactions were highlighted in bold (P values <0.05). Abbreviation: GC, gastric cancer; GA, atrophic gastritis; CON, healthy controls.

**Table 2:** Two-way interaction effect between *PTPN11* tagSNPs and *IL1B* and *TLR4* tagSNPs on risks of gastric cancer and atrophic gastritis

*PGC* tagSNP	*IL1B* rs1143623		*IL1B* rs1143627		*IL1B* rs1143643		*TLR4* rs10983755		*TLR4* rs11536878	
	GG	GC/CC	TT	TC/CC	AA	AG/GG	GG	GA/AA	CC	CA/AA
**For GC vs. CON**										
*PTPN11* rs12229892										
GG	128/87	263/135	101/66	287/154	111/62	280/260	167/115	164/100	315/175	74/42
	1(ref)	0.69(0.47,1.02)	1(ref)	0.70(0.47,1.06)	1(ref)	0.97(0.64,1.45)	1(ref)	0.72(0.50,1.06)	1(ref)	0.97(0.61,1.55)
GA/AA	321/173	553/313	239/129	630/354	241/132	630/356	364/240	358/231	693/383	180/96
	0.74(0.51,1.07)	0.84(0.60,1.18)	0.72(0.47,1.10)	0.81(0.56,1.18)	0.94(0.62,1.43)	1.02(0.71,1.49)	0.93(0.68,1.28)	0.82(0.59,1.12)	1.03(0.80,1.31)	0.97(0.69,1.36)
	**P for interaction=0.034, interaction index=1.64**		P for interaction=0.066, interaction index=1.59		P for interaction=0.634, interaction index=1.13		P for interaction=0.397, interaction index=1.22		P for interaction=0.917, interaction index=0.97	
**For GA vs. CON**										
*PTPN11* rs12229892										
GG	128/103	263/181	101/84	287/200	111/83	280/202	167/144	164/122	315/220	74/63
	1(ref)	0.84(0.59,1.18)	1(ref)	0.76(0.53,1.10)	1(ref)	0.96(0.66,1.37)	1(ref)	0.83(0.59,1.17)	1(ref)	1.29(0.86,1.93)
GA/AA	321/207	553/411	239/154	630/460	241/154	630/464	364/286	358/290	693/501	180/116
	0.77(0.55,1.07)	0.86(0.63,1.18)	0.70(0.48,1.02)	0.78(0.55,1.09)	0.80(0.55,1.17)	0.94(0.67,1.31)	0.85(0.64,1.14)	0.83(0.62,1.11)	0.99(0.80,1.24)	0.90(0.66,1.23)
	P for interaction=0.165, interaction index=1.34		P for interaction=0.090, interaction index=1.47		P for interaction=0.369, interaction index=1.22		P for interaction=0.424, interaction index=1.19		P for interaction=0.167, interaction index=0.71	

All tests were adjusted by age, sex and *H. pylori* infection. Statistically significant interaction was highlighted in bold (P values <0.05). Abbreviation: GC, gastric cancer; GA, atrophic gastritis; CON, healthy controls.

### Epistatic effects of two-way interactions

Among the four polymorphisms involved in significant pairwise interactions, *PGC* rs6912200, *PTPN11* rs12229892 and *IL1B* rs1143623 had no overall main effect on disease risk [7, 17]. We therefore examined the epistatic effects between pairs of interacting factors (Table 3). For *PGC* rs6912200 and *PTPN11* rs12229892, TC/TT genotypes at rs6912200 and GA/AA genotypes at rs12229892 each conferred a reduced risk of gastric cancer and atrophic gastritis, but not if they were present together. For *PGC* rs4711690 and *IL1B* rs1143623, rs4711690 GG/GC genotypes were associated with a reduced risk of gastric cancer and atrophic gastritis, but only in the presence of GC/CC genotypes at rs1143623. For *PTPN11* rs12229892 and *IL1B* rs1143623, rs1143623 GC/CC genotypes were associated with a reduced gastric cancer risk only in the absence of rs12229892 GA/AA genotypes. These observations suggest that *PGC* rs6912200, *PTPN11* rs12229892, and *IL1B* rs1143623 individually have no main effect but demonstrate pairwise epistatic interactions.

**Table 3:** Epistatic effect of pair-wise interacting factors on the risks of gastric cancer and atrophic gastritis

Interacted pair-wise SNPs	Comparison	Subset	GA vs. CON		GC vs. CON	
			OR(95%CI)	P	OR(95%CI)	P
*PGC* rs6912200 interacted with *PTPN11* rs12229892	*PGC* rs6912200 TT/TC vs. CC	*PTPN11* rs12229892 GG	**0.65(0.43,0.99)**	**0.043**	**0.56(0.35,0.90)**	**0.016**
		*PTPN11* rs12229892 AA/GA	1.32(0.99,1.76)	0.059	1.12(0.83,1.51)	0.451
	*PTPN11* rs12229892 AA/GA vs. GG	*PGC* rs6912200 CC	**0.50(0.32,0.79)**	**0.003**	**0.59(0.37,0.94)**	**0.027**
		*PGC* rs6912200 TT/TC	1.00(0.77,1.29)	0.982	1.11(0.84,1.46)	0.466
*PGC* rs4711690 interacted with *IL1B* rs1143623	*PGC* rs4711690 GG/GC vs CC	*IL1B* rs1143623 GG	0.81(0.59,1.10)	0.176	1.07(0.77,1.50)	0.683
		*IL1B* rs1143623 GC/CC	**0.76(0.60,0.96)**	**0.021**	**0.69(0.53,0.90)**	**0.006**
	*IL1B* rs1143623 GC/CC vs GG	*PGC* rs4711690 CC	1.05(0.82,1.35)	0.690	1.18(0.89,2.56)	0.254
		*PGC* rs4711690 GG/GC	0.99(0.73,1.34)	0.946	0.76(0.55,1.06)	0.102
*PTPN11* rs12229892 interacted with *IL1B* rs1143623	*PTPN11* rs12229892 AA/GA vs. GG	*IL1B* rs1143623 GG	0.76(0.55,1.07)	0.114	0.74(0.52,1.07)	0.110
		*IL1B* rs1143623 GC/CC	1.03(0.80,1.31)	0.837	1.23(0.93,1.62)	0.153
	*IL1B* rs1143623 GC/CC vs GG	*PTPN11* rs12229892 GG	0.84(0.60,1.17)	0.295	**0.68(0.47,1.00)**	**0.050**
		*PTPN11* rs12229892 AA/GA	1.11(0.88,1.41)	0.369	1.14(0.88,1.47)	0.331

All tests were adjusted by age, sex and *H. pylori* infection. Statistically significant associations were highlighted in bold (P values <0.05). Abbreviation: GC, gastric cancer; GA, atrophic gastritis; CON, healthy controls.

### Interactions involving multiple polymorphisms of PGC, PTPN11, and IL1B genes

Next, we explored potential three- and four-way interactions among the four polymorphisms involved in significant pairwise interactions. A three-way interaction between *PGC* rs4711690 CG/GG, *PGC* rs6912200 CT/TT, and *PTPN11* rs12229892 GA/AA was significantly associated with atrophic gastritis risk (*P* value for interaction = 0.048, interaction index = 2.82) (Table 4). We analyzed the ORs by dividing the combined population into four subgroups based on the number of interacting genotypes (Table 5). A significant dosage effect was observed, with an increasing number of protective genotypes being associated with a decreasing risk of atrophic gastritis (P_trend_ = 0.005). Four-way interactions among the four SNPs in relation to the risks of gastric cancer or atrophic gastritis did not reach statistical significance (Supplementary Table 5).

**Table 4:** Three-way interaction effect of *PGC* rs4711690, *PGC* rs6912200 and *PTPN11* rs12229892 on atrophic gastritis risk

*PGC*	*PGC*	*PTPN11*	CON (n)	GA (n)	GA vs CON	
rs4711690	rs6912200	rs12229892			OR(95%CI)	P
**Total population^a^**						
CC	CC	GG	22	26	1(ref)	
CC	CC	GA/AA	46	46	0.83(0.41,1.67)	0.596
CC	CT/TT	GG	135	131	0.80(0.43,1.48)	0.47
CC	CT/TT	GA/AA	271	251	0.77(0.42,1.39)	0.38
CG/GG	CC	GG	33	45	1.15(0.56,2.38)	0.707
CG/GG	CC	GA/AA	121	81	0.55(0.29,1.03)	0.062
CG/GG	CT/TT	GG	82	44	0.44(0.23,0.87)	0.018
CG/GG	CT/TT	GA/AA	169	140	0.69(0.38,1.28)	0.238
					**P for interaction=0.048, interaction index=2.82**	
***H. pylori-*negative subpopulation^b^**						
CC	CC	GG	14	17	1(ref)	
CC	CC	GA/AA	29	13	0.35(0.13,0.91)	0.032
CC	CT/TT	GG	103	71	0.54(0.25,1.17)	0.116
CC	CT/TT	GA/AA	199	106	0.41(0.20,0.88)	0.021
CG/GG	CC	GG	22	15	0.56(0.21,1.48)	0.241
CG/GG	CC	GA/AA	88	27	0.24(0.10,0.54)	0.001
CG/GG	CT/TT	GG	55	19	0.27(0.11,0.65)	0.004
CG/GG	CT/TT	GA/AA	121	37	0.24(0.11,0.54)	<0.001
					P for interaction=0.960, interaction index=0.96	
***H. pylori*-positive subpopulation^b^**						
CC	CC	GG	8	9	1(ref)	
CC	CC	GA/AA	17	33	1.83(0.59,5.66)	0.294
CC	CT/TT	GG	32	60	1.71(0.60,4.91)	0.32
CC	CT/TT	GA/AA	72	145	1.92(0.70,5.24)	0.203
CG/GG	CC	GG	27	25	2.50(0.76,8.19)	0.131
CG/GG	CC	GA/AA	48	103	1.51(0.53,4.35)	0.444
CG/GG	CT/TT	GG	17	9	0.86(0.29,2.61)	0.794
CG/GG	CT/TT	GA/AA	28	17	2.05(0.74,5.70)	0.17
					**P for interaction=0.029, interaction index=6.13**	

a , these tests were adjusted by age, sex and *H. pylori* infection;

b , these tests were adjusted by age and sex. Statistically significant interactions were highlighted in bold (P values <0.05). Abbreviation: GA, atrophic gastritis; CON, healthy controls.

**Table 5:** Cumulative effect of the three interacting factors of *PGC* rs6912200, *PGC* rs4711690 and *PTPN11* rs12229892 on the risk of atrophic gastritis

No. of interacting genotypes	Total population			*H. pylori*-negative subpopulation			*H. pylori*-positive subpopulation			Pbd^c^
	Controls/cases	OR(95%CI)	P^a^	Controls/cases	OR(95%CI)	P^b^	Controls/cases	OR(95%CI)	P^b^	
0	22/26	1(ref)		14/17			8/9			
1	214/222	0.76(0.40,1.43)	0.394	154/99	0.51(0.24,1.08)	0.078	60/126	1.87(0.68,5.15)	0.226	**0.049**
2	474/376	0.56(0.30,1.04)	0.068	342/152	0.35(0.17,0.72)	0.005	132/224	1.60(0.60,4.29)	0.353	**0.020**
3	169/140	0.54(0.28,1.03)	0.062	121/37	0.24(0.11,0.54)	4.96×10^−4^	48/103	2.02(0.73,5.63)	0.179	**0.001**
		**P trend=0.005**								

a , these tests were adjusted by sex, age and *H. pylori* infection;

b , these tests were adjusted by sex and age;

c , Breslow-Day test was employed to assess the homogeneity of stratum-specific ORs between *H. pylori*-negative and -positive subpopulations. Statistically significant results were highlighted in bold (P values <0.05). Abbreviation: GC, gastric cancer; GA, atrophic gastritis; CON, healthy controls.

### Effect modification of *H. pylori* infection on the interaction between *PGC* rs4711690, *PGC* rs6912200, and *PTPN11* rs12229892

Intriguingly, all loci involved in the significant three-way interaction detailed above have also been shown to have interaction effects with *H. pylori* infection, as previously described [7, 17]. We therefore felt that it was important to evaluate whether *H. pylori* infection modifies the effect of this three-way genetic interaction on the risk of atrophic gastritis. We first tested the effect modification of *H. pylori* on the interaction strength in a stratified analysis according to *H. pylori* infection status (Table 4). The interaction index was 0.96 in the *H. pylori*-negative subpopulation (*P* = 0.960), whereas it was 6.13 in the *H. pylori-*positive subpopulation (*P* = 0.029), suggesting that the interaction effect on atrophic gastritis risk is restricted to the cases infected with *H. pylori*.

We further tested the effect modification of *H. pylori* on the cumulative effect of the three interacting SNPs. We used the Breslow-Day test to compare the differences between the ORs of each comparison in *H. pylori-*negative and -positive subgroups (Table 5). The subjects with one, two, or three variant genotypes had significantly different atrophic gastritis risks between the two subgroups based on *H. pylori* infection status (*P* value for Breslow-Day test = 0.049, 0.020, and 0.001, respectively), suggesting that *H. pylori* infection can modify the cumulative effect of the three interacting SNPs.

## DISCUSSION

Gastric cancer is presumed to be the cumulative result of interactions among many genes, with each gene only having a small effect. In this study, we found new SNP interactions among *H. pylori-*related host genes *PGC*, *PTPN11*, and *IL1B* modifying the susceptibility to atrophic gastritis and gastric cancer. When we considered the host genetic effects alone, gene–gene interactions consistently contributed to reduced risks of gastric cancer and/or atrophic gastritis, including two-way interactions: *PGC* rs6912200-*PTPN11* rs12229892, *PGC* rs4711690-*IL1B* rs1143623 and *PTPN11* rs12229892-*IL1B* rs1143623 and a three-way interaction: *PGC* rs4711690- *PGC* rs6912200-*PTPN11* rs12229892. Interestingly, when the effect modification of *H. pylori* infection was evaluated, the cumulative effect of the three-way interaction of *PGC* rs4711690- *PGC* rs6912200-*PTPN11* rs12229892 was shown to differ by the status of *H. pylori* infection. The cumulative effects on atrophic gastritis susceptibility switched from being beneficial to being risky in the presence of *H. pylori* infection.

Since 2008, genome wide association studies (GWAS) have been performed to search for gastric cancer susceptibility loci [18], and several associated regions such as 1q22, 3q13.31, 5p13.1, 6p21.1, 8q24, 10q23, and 20p13 have been revealed [18-26]. However, fine-mapping susceptibility loci within these regions is still required. In this study, we selected four important host genes involving in the response to *H. pylori* infection. Among them, *PGC* gene that plays an important role in gastric epithelial differentiation is located at 6p21.1. This region has been revealed to be an important susceptibility loci for multiple cancers such as noncardia gastric cancer, lung cancer, and esophageal squamous-cell carcinoma in a Chinese GWAS [25]. Currently, we focused on gene-gene interaction effect instead of individual gene effect, and *PGC* was observed to have interaction effect with *PTPN11* at 12q24 and *IL1B* at 2q14 in susceptibility of gastric carcinogenesis. The interaction effect on atrophic gastritis risk between *PGC* rs6912200 and *PTPN11* rs12229892 (OR = 0.60) was greater than the main effect of a single polymorphism, *PGC* rs4711690 (OR = 0.78) that was identified in our previous study [7]. Moreover, there was a three-way interaction, *PGC* rs4711690-*PGC* rs6912200-*PTPN11* rs12229892, whose beneficial effect increased cumulatively with each additional SNP (OR = 0.73, 0.56, and 0.54 for one, two, and three interacting variant SNPs, respectively). The cumulative effect of the three SNPs was stronger than the effect of each SNP alone, which is indicative of a true interaction.

Notably, among the four significant interacting polymorphisms, only *PGC* rs4711690 was previously found to have a main effect on disease risk while *PGC* rs6912200, *PTPN11* rs12229892, and *IL1B* rs1143623 had no such effect [7, 17]. Indeed, such an interaction effect between polymorphisms of two or more genes in the absence of a significant main effect of any of them, is indicative of epistasis [27]. As such, the genetic effects of the *PGC* rs6912200, *PTPN11* rs12229892, and *IL1B* rs1143623 polymorphisms on disease risks would have been missed had they not been tested jointly. Multiple studies have shown that epistatic gene-gene interactions confer susceptibility to various malignancies, such as breast cancer, lung cancer, and colorectal cancer [28-30]. In fact, only in rare cases does the disease appear to be monogenic and, generally, multiple genes are involved in tumor initiation and development. This addresses, in part, the apparent missing heritability of gastric cancer risk and provides novel insights into the multifactorial etiology of gastric cancer.

In the current study, the most significant epistatic gene-gene effect was between *PGC* rs6912200 and *PTPN11* rs12229892. Both TC/TT genotypes at rs6912200 and GA/AA genotypes at rs12229892 conferred a reduced risk of gastric cancer and atrophic gastritis in the absence of the other variant SNP, but showed no effect in its presence. In another epistatic interaction, the *IL1B* rs1143623 GC/CC genotypes showed an association with a reduced gastric cancer risk only if the *PTPN11* rs12229892 GA/AA genotypes were absent. These observations suggest that the effects of *PGC* rs6912200, *PTPN11* rs12229892, and *IL1B* rs1143623 on gastric cancer development principally rely on the status of the other SNP in each pair-wise interaction. However, the evidence for a direct functional relationship between the alleles of *PGC*, *PTPN11*, and *IL1B* is scarce. Nonetheless, it is possible to speculate that they interact with one another via various signal transduction pathways and our interactions may reflect this. For instance, the IL1B cytokine and Shp2 factor (PTPN11) could mutually activate each other through their related ERK and MAPK pathways [9-11, 31]. Activation of the ERK pathway could then promote the expression of the PGC protein [32]. Additionally, Shp2 has a central role in several other pathways coordinating various cellular processes in response to extracellular stimuli, including those affecting cell growth and motility [33]. Genetic variants of any gene within these networks could potentially have an effect on the action of the other genes and could thus disturb the balance of homeostasis of gastric epithelial cells. Since we only included tagSNPs of the genes of interest in this study, other functional SNPs covered by the tagSNPs but not involved in this study yet could also participate in the interaction. Further independent study that covered more SNPs in addition to tagSNPs are warranted, and comprehensive function experiment involved two or more genes would be informative to estimate the role of susceptibility loci that directly affect gastric cancer development.

The phenomenon of an effect modification by *H. pylori* infection observed in the *PTPN11* and *PGC* interaction may provide an important hint to help prevent gastric cancer by eradicating *H. pylori* in susceptible people. Of importance, *PTPN11* and *PGC* function as critical host genes in the network of *H. pylori* pathogenicity in the gastric epithelium [8-14]. One of the most important virulence factors of *H. pylori*, CagA, can activate the *PTPN11* encoded protein, Shp2, and its related MAPK pathways and induce epithelial transformation, proliferation, and inflammation [34]. PGC protein acts as a critical gastric effector of signals stimulated by the LPS of CagA (+) *H. pylori* [35]. The effect modification of *H. pylori* on such host genes might be ubiquitous in the stomach but has been ignored in many studies. We previously found that each of *PGC* rs4711690, *PGC* rs6912200, and *PTPN11* rs12229892 had an interaction effect with *H. pylori* [7]. In the current study, significant effect heterogeneity by *H. pylori* infection status was also observed for the three-way interaction of *PGC* rs4711690, *PGC* rs6912200, and *PTPN11* rs12229892. Moreover, the interaction strength was found to be enhanced in the *H. pylori-*infected subpopulation. This phenomenon indicated an effect modification by *H. pylori* on the cumulative effect of interacting host factors. It is plausible that *H. pylori* may function as a bridge for SNP–SNP interactions between the *PTPN11* and *PGC* genes, in which certain virulence factors or modulins of this microbe may modify the host gene's innate function. Despite previous efforts, the mechanism by which *H. pylori* interacts with each polymorphism remains elusive. Further functional research concerning the role of *H. pylori* in *PGC* and *PTPN11* interactions is warranted, which may, in part, compensate for the probability of false positive/negative findings.

Due to a retrospective study design, the data of enrolled subjects' basic characteristic were also retrospectively extracted from registered databank. However, some information of a portion of the enrolled subjects was lacked, such as smoking and drinking status, family history, and economic status. Accordingly, when we measured the association strength, only the status of sex, age and *H. pylori* infection were adjusted, which may be a main limitation of the current study. Therefore, more potential confounding factors should be included in further independent replication study.

In summary, we observed novel SNP interactions among *PGC*, *PTPN11*, and *IL1B* which modified the risks of gastric cancer and atrophic gastritis and we provided important hints of effect modification by *H. pylori* infection on the cumulative effect of *PGC* rs6912200, *PGC* rs4711690, and *PTPN11* rs12229892. Better understanding the gene–gene and gene–environment interactions could provide important insights into the etiology of gastric cancer. The potential impact of *H. pylori* infection on genetic susceptibility in the prediction and prevention of gastric cancer needs to be considered in future studies.

## MATERIALS AND METHODS

### Study population

This study was approved by the Human Ethics Review Committee of First Affiliated Hospital of China Medical University. Written informed consent was obtained from each participant. A total of 2897 subjects consisting of 1276 healthy controls, 907 cases of atrophic gastritis, and 714 cases of gastric cancer were included in the current study. A full description of the inclusion criteria, diagnostic criteria, and characteristics of the study population has been reported previously [7, 17]. Briefly, all the subjects were Chinese and living in northern China. They were recruited between 2002 and 2011 from a health check program for gastric cancer screening or from hospitals in Zhuanghe or Shenyang in Liaoning Province, China. The diagnoses of all the study subjects were independently made by two gastrointestinal pathologists according to the Consensus on Chronic Gastritis formulated at the National Symposium in combination with the updated Sydney System and the World Health Organization (WHO) criteria [36-38]. The healthy control subjects in the current study comprised individuals with normal stomachs or with only slight superficial gastritis without atrophic lesions or intestinal metaplasia. Subjects who had a history of other malignant tumors were excluded.

### SNP selection and genotyping

As described in our previous studies [7, 17], we employed a two-step approach to select tagSNPs for the genes of interest. Briefly, Haploview software (http://www.broadinstitute.org/mpg/haploview) was used to minimize the number of SNPs that needs to be genotyped and FastSNP search (http://FastSNP.ibms.sinica.edu.tw/) was performed to predict their functional effects. The predicted function of each tagSNP selected in this study were summarized in Supplementary table 1. Genomic DNA was isolated from peripheral blood lymphocytes by the routine phenol–chloroform method. The genotypes of 13 tagSNPs (rs4711690, rs6458238, rs9471643, rs6941539, rs6912200, rs3789210, and rs6939861 of *PGC*; rs10983755 and rs11536878 of *TLR4*; rs12229892 of *PTPN11*; and rs1143623, rs1143627, and rs1143643 of *IL1B*) were selected and assessed by the Sequenom MassARRAY platform (Sequenom, San Diego, CA, USA) according to the manufacturer's instructions [7, 17]. Each DNA sample was diluted to a working concentration of 50 ng/μL for genotyping. All samples were randomly placed on the 384-well plates and the operator confirming the SNP genotyping calls was blinded to disease status. Randomly selected samples had repeat genotypes performed and 100% concordance was confirmed.

### ELISA assessment of H. pylori immunoglobulin G (IgG) in serum

Serum *H. pylori* IgG levels were determined by enzyme-linked immunosorbent assay (*H. pylori* IgG ELISA kit, BIOHIT, Helsinki, Finland). A reading > 34 enzyme immune units was defined to be *H. pylori* seropositive.

### Statistical analysis

We set the combination of common genotypes as the reference and employed the likelihood-ratio test to assess the SNP–SNP interaction effects by comparing the model that only contained the main effects of each factor with the full model that also contained the interaction terms. Odds ratios (ORs) with their 95% confidence intervals (CI) were calculated as measures of associations adjusted by sex, age and *H. pylori* infection unless the *H. pylori* has been used as a stratified factor. The Cochrane-Armitage test for linear trend was used to examine whether there was a dosage effect on disease risk with an increasing number of interacting SNP genotypes. To compare the effect modification of *H. pylori* status on the cumulative effect of the interacting SNPs on disease risk, the Breslow-Day test was employed to assess the homogeneity of stratum-specific ORs across different subgroups. All the analyses were performed using SPSS 16.0 software (SPSS Inc., Chicago, IL, USA) and Stata version 11.0 (StataCorp., College Station, TX, USA). All *P* values were two sided, and *P* values < 0.05 were considered statistically significant.

## SUPPLEMENTARY TABLES



## Abbreviations

PGC: pepsinogen C
PTPN11: protein tyrosine phosphatase, non-receptor type 11
TLR4: Toll-like receptor 4
IL1B: interleukin-1B
H. pylori: Helicobacter pylori
LPS: lipopolysaccharide
CagA: cytotoxin-associated antigen A
NF-Kb: nuclear factor-kappa B
MAPK: mitogen-activated protein kinase
SNP: single nucleotide polymorphism
WHO: World Health Organization
IgG: immunoglobulin G
OR: Odds ratio
CI: confidence interval
