# Supplementary material for: SNP interactions of Helicobacter pylori-related host genes PGC, PTPN11, IL1B, and TLR4 in susceptibility to gastric carcinogenesis
Source: Oncotarget. 2015 May 22;6(22):19017–26. doi: 10.18632/oncotarget.4231 (PMC4662472; doi:10.18632/oncotarget.4231)
Supplement: Supplementary file 1 [file oncotarget-06-19017-s001.pdf]

SNP interactions of *Helicobacter pylori*-related host genes *PGC, PTPN11, IL1B,* and *TLR4* in susceptibility to gastric carcinogenesis

Supplementary Material

Supplementary Table 1: Predicted function of the selected tagSNPs in the four genes of interest

Gene symbol	tagSNP_ID, allele	Position	Predicted functional effects[a]	Tagging SNP
TLR4	rs10983755,G/A	upstream	at TF binding site	rs10983755,rs10759932
	rs11536878,A/C	intron	intronic enhancer	rs11536878,rs11536879
PTPN11	rs12229892,A/G	intron	at TF binding site	rs12229892
IL1B	rs1143643,A/G	intron	splicing regulation	rs1143643,rs3917368,rs1143633
	rs1143627,C/T	upstream	at TF binding site	rs1143627,rs16944,rs10169916,rs4848306, rs13013349,rs13032029
	rs1143623,C/G	UTR	at TF binding site	rs1143623,rs12621220
PGC	rs4711690,C/G	intron	splicing regulation	rs4711690,rs4714509,rs3804279,rs12664901
	rs9471643,G/C	upstream	at TF binding site	rs9471643
	rs6458238,A/G	upstream	at TF binding site	rs6458238,rs11967238
	rs6912200,C/T	upstream	no known function	rs6912200,rs6907935
	rs6939861,A/G	downstream	no known function	rs6939861
	rs3789210,C/G	intron	no known function	rs3789210
	rs6941539,C/T	upstream	no known function	rs6941539,rs6914124

[a], The functional effects for the selected tagSNPs were predicted using the FastSNP Search tool (http://FastSNP.ibms.sinica.edu.tw/).

Supplementary Table 2: Two-way interaction effect between PGC tagSNP and TLR4 and IL1B tagSNP on gastric cancer risk

PGC tagSNP		IL1B rs1143643		IL1B rs1143627		TLR4 rs10983755		TLR4 rs11536878	
		AA	AG/GG	TT	TC/CC	GG	GA/AA	CC	CA/AA
rs4711690									
CC	Controls/Cases	202/110	501/311	192/112	510/304	286/217	294/188	566/324	138/92
	OR(95%CI)	1(ref)	1.20(0.89,1.62)	1(ref)	1.11(0	1(ref)	0.76(0.57,1.00)	1(ref)	1.14(0.82,1.58)
CG/GG	Controls/Cases	150/84	408/205	149/82	82,1.50)	244/136	226/144	442/231	116/47
	OR(95%CI)	1.02(0.69,1.51)	0.90(0.66,1.24)	1.03(0.69,1.52)	0.84(0.61,1.16)	0.71(0.53,0.96)	0.68(0.50,0.92)	0.87(0.69,1.10)	0.63(0.42,0.95)
		P for interaction=0.793, interaction index =0.74		P for interaction=0.207, interaction index =0.74		P for interaction=0.272, interaction index =1.27		P for interaction=0.099, interaction index =0.64	
rs6458238									
GG	Controls/Cases	281/164	755/422	274/161	758/418	435/300	431/266	836/456	201/116
	OR(95%CI)	1(ref)	0.98(0.76,1.26)	1(ref)	0.91(0.70,1.17)	1(ref)	0.79(0.63,1.00)	1(ref)	1.03(0.77,1.37)
AG/AA	Controls/Cases	71/30	157/95	67/35	159/91	97/56	90/65	175/103	52/23
	OR(95%CI)	0.68(0.41,1.14)	1.01(0.71,1.43)	0.73(0.44,1.21)	0.94(0.66,1.34)	0.85(0.58,1.27)	0.89(0.61,1.30)	1.03(0.76,1.38)	0.73(0.42,1.28)
		P for interaction=0.181, interaction index =1.50		P for interaction=0.246, interaction index =1.42		P for interaction=0.328, interaction index =1.32		P for interaction=0.284, interaction index =0.70	
rs9471643									
GG/CC	Controls/Cases	215/124	554/335	208/129	561/323	331/219	316/223	620/357	150/91
	OR(95%CI)	1(ref)	1.15(0.86,1.53)	1(ref)	1.02(0.76,1.35)	1(ref)	0.92(0.70,1.20)	1(ref)	0.98(0.71,1.35)

GC	Controls/Cases	138/70	352/181	132/66	357/185	200/136	204/108	389/200	102/48
	OR(95%CI)	0.91(0.61,1.36)	0.85(0.82,1.16)	0.87(0.58,1.30)	0.78(0.57,1.07)	0.91(0.67,1.24)	0.63(0.46,0.86)	0.80(0.63,1.01)	0.75(0.50,1.12)
		P for interaction=0.389, interaction index =0.81		P for interaction=0.613, interaction index =0.88		P for interaction=0.194, interaction index =0.75		P for interaction=0.882, interaction index =0.96	
rs3789210									
CC	Controls/Cases	178/126	491/360	178/133	489/349	346/252	325/235	543/388	125/93
	OR(95%CI)	1(ref)	0.98(0.74,1.31)	1(ref)	0.93(0.70,1.24)	1(ref)	0.86(0	1(ref)	0.97(0.70,1.33)
GC/GG	Controls/Cases	68/50	152/105	65/40	154/113	105/78	66,1.10)	176/118	44/31
	OR(95%CI)	0.86(0.54,1.37)	0.98(0.69,1.41)	0.73(0.45,1.19)	0.98(0.69,1.40)	0.94(0.65,1.35)	0.83(0.58,1.19)	0.94(0.70,1.25)	0.90(0.53,1.51)
		P for interaction=0.602, interaction index =1.16		P for interaction=0.213, interaction index =1.44		P for interaction=0.891, interaction index =1.04		P for interaction=0.981, interaction index =0.99	
rs6912200									
CC	Controls/Cases	59/36	166/129	55/43	168/119	116/81	110/85	184/134	42/28
	OR(95%CI)	1(ref)	1.49(0.90,2.47)	1(ref)	0.83(0.50,1.36)	1(ref)	0.92(0.60,1.41)	1(ref)	0.86(0.49,1.51)
CT/TT	Controls/Cases	185/138	476/335	187/129	473/341	332/249	330/223	532/372	128/96
	OR(95%CI)	1.33(0.80,2.19)	1.22(0.76,1.94)	0.76(0.46,1.24)	0.82(0.52,1.30)	0.97(0.68,1.37)	0.80(0.56,1.14)	0.92(0.70,1.22)	0.91(0.63,1.32)
		P for interaction=0.100, interaction index =0.62		P for interaction=0.352, interaction index OR=1.32		P for interaction=0.685, interaction index =0.90		P for interaction=0.679, interaction index =1.15	
rs6939861									
GG	Controls/Cases	106/65	283/181	99/68	286/175	208/125	181/120	318/194	69/46
	OR(95%CI)	1(ref)	1.04(0.71,1.53)	1(ref)	0.86(0.58,1.27)	1(ref)	0.97(0.69,1.36)	1(ref)	0.92(0.59,1.43)
AG/AA	Controls/Cases	129/102	327/272	135/99	321/272	221/198	238/177	364/298	94/73
	OR(95%CI)	1.32(0.86,2.03)	1.39(0.96,2.02)	1.04(0.67,1.60)	1.24(0.85,1.80)	1.50(1.09,2.05)	1.13(0.82,1.55)	1.33(1.04,1.71)	1.29(0.88,1.88)

		P for interaction=0.970, interaction index =1.01		P for interaction=0.205, interaction index =1.39		P for interaction=0.279, interaction index =0.78		P for interaction=0.857, interaction index =1.05	
rs6941539									
CC	Controls/Cases	179/131	459/329	178/124	456/334	333/240	306/219	513/362	122/91
	OR(95%CI)	1(ref)	1.00(0.75,1.33)	1(ref)	1.10(0.83,1.48)	1(ref)	0.90(0.70,1.17)	1(ref)	0.96(0.70,1.34)
CT/TT	Controls/Cases	65/47	180/133	62/51	184/125	116/92	130/89	201/142	46/31
	OR(95%CI)	1.02(0.64,1.63)	1.07(0.76,1.51)	1.34(0.83,2.15)	1.02(0.72,1.45)	1.17(0.83,1.65)	0.87(0.62,1.23)	1.03(0.78,1.35)	0.96(0.57,1.60)
		P for interaction=0.853, interaction index=1.05		P for interaction=0.194, interaction index =0.69		P for interaction=0.453, interaction index =0.83		P for interaction=0.918, interaction index =0.97	

All tests were adjusted by age, sex and *H. pylori* infection.

Supplementary Table 3: Two-way interaction effect between PGC tagSNP and TLR4 and IL1B tagSNP on atrophic gastritis risk

PGC		IL1B rs1143643		IL1B rs1143627		TLR4 rs10983755		TLR4 rs11536878	
tagSNP		AA	AG/GG	TT	TC/CC	GG	GA/AA	CC	CA/AA
rs4711690									
CC	Control/Case	202/145	501/393	192/146	510/389	286/260	294/245	566/424	138/117
	OR(95%CI)	1(ref)	1.09(0.84,1.43)	1(ref)	0.97(0.74,1.27)	1(ref)	0.89(0.69,1.15)	1(ref)	1.22(0.91,1.64)
CG/GG	Control/Case	150/92	408/269	149/91	405/269	244/169	226/166	442/396	116/62
	OR(95%CI)	0.78(0.54,1.12)	0.85(0.64,0.13)	0.74(0.52,1.06)	0.78(0.59,1.03)	0.70(0.53,0.92)	0.69(0.52,0.92)	0.85(0.69,1.04)	0.65(0.45,0.93)
		P for interaction=0.998, interaction index=1.00		P for interaction=0.699, interaction index=1.09		P for interaction=0.551, interaction index=1.13		P for interaction=0.054, interaction index=0.63	
rs6458238									
GG	Control/Case	281/202	755/570	274/204	758/563	435/362	431/361	836/622	201/148
	OR(95%CI)	1(ref)	1.05(0.84,1.31)	1(ref)	0.94(0.75,1.18)	1(ref)	0.98(0.79,1.21)	1(ref)	1.01(0.79,1.30)
AG/AA	Control/Case	71/35	157/96	67/34	159/98	97/67	90/53	175/101	52/31
	OR(95%CI)	0.64(0.40,1.02)	0.81(0.58,1.13)	0.58(0.36,0.94)	0.76(0.54,1.05)	0.87(0.61,1.25)	0.62(0.42,0.92)	0.72(0.54,0.95)	0.81(0.50,1.31)
		P for interaction=0.497, interaction index=1.21		P for interaction=0.254, interaction index=1.39		P for interaction=0.255, interaction index=0.73		P for interaction=0.719, interaction index=1.12	
rs9471643									
GG/CC	Control/Case	215/143	554/426	208/150	561/414	330/260	316/267	620/448	150/121
	OR(95%CI)	1(ref)	1.14(0.88,1.48)	1(ref)	1.02(0.79,1.33)	1(ref)	1.03(0.81,1.32)	1(ref)	1.18(0.88,1.57)

GC	Control/Case	138/93	352/237	132/87	357/243	200/168	204/145	389/272	102/57
	OR(95%CI)	0.93(0.65,1.33)	0.96(0.72,1.28)	0.92(0.64,1.33)	0.87(0.66,1.16)	1.00(0.75,1.32)	0.81(0.61,1.07)	0.93(0.76,1.15)	0.73(0.50,1.06)
		P for interaction=0.647, interaction index=0.91		P for interaction=0.718, interaction index=0.92		P for interaction=0.209, interaction index=0.77		P for interaction=0.096, interaction index=0.67	
rs3789210									
CC	Control/Case	178/163	491/445	178/162	489/442	346/314	325/297	543/494	125/115
	OR(95%CI)	1(ref)	1.29(0.75,2.21)	1(ref)	0.93(0.72,1.22)	1(ref)	0.97(0.77,1.22)	1(ref)	1.04(0.77,1.39)
GC/GG	Control/Case	68/48	152/114	65/51	154/111	105/80	115/81	176/131	44/31
	OR(95%CI)	0.95(0.73,1.23)	0.65(0.42,1.03)	0.78(0.50,1.23)	0.74(0.52,1.04)	0.81(0.57,1.14)	0.72(0.51,1.02)	0.80(0.61,1.04)	0.75(0.45,1.24)
		P for interaction=0.353, interaction index=1.29		P for interaction=0.987, interaction index=1.00		P for interaction=0.760, interaction index=0.93		P for interaction=0.749, interaction index=0.90	
rs6912200									
CC	Control/Case	59/62	166/136	55/58	168/140	116/95	110/103	184/160	42/37
	OR(95%CI)	1(ref)	0.78(0.50,1.22)	1(ref)	0.77(0.48,1.21)	1(ref)	1.10(0.73,1.65)	1(ref)	1.13(0.67,1.89)
CT/TT	Control/Case	185/148	476/420	187/153	473/411	332/298	330/272	532/460	128/109
	OR(95%CI)	0.82(0.52,1.27)	0.90(0.60,1.34)	0.83(0.53,1.31)	0.85(0.56,1.29)	1.17(0.84,1.63)	1.04(0.74,1.45)	1.09(0.84,1.41)	1.05(0.74,1.50)
		P for interaction=0.200, interaction index=1.41		P for interaction=0.289, interaction index=1.33		P for interaction=0.373, interaction index=0.81		P for interaction=0.621, interaction index=0.86	
rs6939861									
GG	Control/Case	106/74	283/221	99/81	286/212	208/161	181/136	318/236	69/60
	OR(95%CI)	1(ref)	1.15(0.79,1.65)	1(ref)	0.88(0.61,1.26)	1(ref)	0.89(0.65,1.23)	1(ref)	1.18(0.78,1.77)
AG/AA	Control/Case	129/128	327/323	135/126	321/323	221/217	238/234	364/371	94/79
	OR(95%CI)	1.50(1.00,2.25)	1.45(1.02,2.07)	1.16(0.77,1.74)	1.20(0.84,1.70)	1.25(0.93,1.68)	1.23(0.92,1.65)	1.39(1.10,1.76)	1.19(0.83,1.72)

		P for interaction=0.496, interaction index=0.85		P for interaction=0.503, interaction index=1.18		P for interaction=0.643, interaction index=1.11		P for interaction=0.253, interaction index=0.73	
rs6941539									
CC	Control/Case	179/142	459/385	178/148	456/376	333/276	306/253	513/419	122/109
	OR(95%CI)	1(ref)	1.08(0.82,1.43)	1(ref)	0.96(0.73,1.26)	1(ref)	0.94(0.73,1.20)	1(ref)	1.08(0.79,1.46)
CT/TT	Control/Case	65/66	180/172	62/63	184/174	116/114	130/124	201/200	46/37
	OR(95%CI)	1.37(0.89,2.11)	1.20(0.87,1.65)	1.22(0.79,1.90)	1.08(0.78,1.49)	1.12(0.81,1.55)	1.12(0.82,1.53)	1.17(0.92,1.51)	1.09(0.68,1.76)
		P for interaction=0.412, interaction index=0.81		P for interaction=0.779, interaction index=0.93		P for interaction=0.778, interaction index=1.07		P for interaction=0.647, interaction index=0.87	

All tests were adjusted by age, sex and *H. pylori* infection.

Supplementary Table 4: Two-way interaction effect between TLR4 tagSNP and IL1B tagSNP on the risks of gastric cancer and atrophic gastritis

TLR4 tagSNP	IL1B rs1143623		IL1B rs1143627		IL1B rs1143643	
	GG	GC/CC	TT	TC/CC	AA	AG/GG
**For GC vs. CON**						
TLR4 rs10983755						
GG	184/136	347/218	142/105	386/247	140/106	392/249
	1(ref)	0.84(0.62,1.15)	1(ref)	0.83(0.60,1.15)	1(ref)	0.87(0.63,1.21)
GA/AA	197/116	326/215	147/85	373/244	157/82	365/250
	0.66(0.46,0.93)	0.79(0.58,1.08)	0.64(0.42,0.95)	0.77(0.55,1.07)	0.62(0.41,0.91)	0.81(0.59,1.13)
	P for interaction=0.255, interaction index=1.40(0.79,2.48)		P for interaction=0.125, interaction index=1.45(0.90,2.33)		P for interaction=0.084, interaction index=1.51(0.95,2.42)	
TLR4 rs11536878						
CC	360/202	650/354	275/156	731/395	273/149	735/408
	1(ref)	0.99(0.78,1.26)	1(ref)	0.95(0.73,1.22)	1(ref)	1.02(0.79,1.32)
CA/AA	90/51	164/88	66/33	186/106	79/37	174/102
	0.94(0.61,1.44)	0.96(0.68,1.36)	0.82(0.49,1.37)	0.98(0.70,1.38)	0.76(0.46,1.23)	1.07(0.76,1.52)
	P for interaction=0.910, interaction index=1.03(0.61,1.76)		P for interaction=0.442, interaction index=1.26(0.70,2.30)		P for interaction=0.255, interaction index=1.40(0.79,2.48)	
**For GA vs. CON**						
TLR4						

rs10983755						
GG	184/143	347/285	142/115	386/312	140/102	392/327
	1(ref)	1.00(0.75,1.33)	1(ref)	0.95(0.70,1.29)	1(ref)	1.14(0.83,1.55)
GA/AA	197/153	326/261	147/114	373/295	157/122	365/290
	0.94(0.68,1.29)	0.94(0.70,1.25)	0.91(0.63,1.32)	0.89(0.66,1.21)	1.01(0.70,1.46)	1.04(0.76,1.43)
	P for interaction=0.959, interaction index=0.99(0.66,1.48)		P for interaction=0.936, interaction index=1.02(0.66,1.57)		P for interaction=0.657, OR interaction index=0.91(0.59,1.40)	
TLR4						
rs11536878						
CC	360/260	650/463	275/199	731/518	273/191	735/529
	1(ref)	0.96(0.77,1.19)	1(ref)	0.96(0.76,1.21)	1(ref)	1.07(0.85,1.34)
CA/AA	90/51	164/128	66/37	186/141	79/46	174/133
	0.79(0.52,1.18)	1.10(0.82,1.49)	0.88(0.55,1.40)	1.04(0.76,1.41)	0.94(0.61,1.45)	1.13(0.83,1.54)
	P for interaction=0.126, interaction index=1.47(0.90,2.40)		P for interaction=0.453, interaction index=1.23(0.72,2.10)		P for interaction=0.648, interaction index=1.13(0.68,1.88)	

All tests were adjusted by age, sex and *H. pylori* infection. Abbreviation: GC, gastric cancer; GA, atrophic gastritis; CON, healthy controls.

Supplementary Table 5: Four-way interaction effect between PGC rs4711690, PGC rs6912200, PTPN11 rs12229892 and IL1B rs1143623 on the risks of gastric cancer and atrophic gastritis

PGC	PGC	PTPN11	IL1B	GC vs CON		GA vs CON	
rs4711690	rs6912200	rs12229892	rs1143623	OR(95%CI)	P	OR(95%CI)	P
CC	CC	GG	GG	1(ref)		1(ref)	
CC	CC	GG	GC/CC	1.34(0.33,5.47)	0.681	0.43(0.12,1.50)	0.186
CC	CC	GA/AA	GG	2.13(0.55,8.20)	0.271	0.37(0.11,1.28)	0.118
CC	CC	GA/AA	GC/CC	1.07(0.30,3.81)	0.915	0.37(0.12,1.10)	0.073
CC	CT/TT	GG	GG	1.01(0.29,3.46)	0.994	0.45(0.15,1.31)	0.142
CC	CT/TT	GG	GC/CC	0.91(0.28,2.97)	0.873	0.49(0.18,1.35)	0.166
CC	CT/TT	GA/AA	GG	0.77(0.23,2.50)	0.656	0.38(0.14,1.03)	0.058
CC	CT/TT	GA/AA	GC/CC	1.37(0.43,4.38)	0.591	0.43(0.16,1.15)	0.093
CG/GG	CC	GG	GG	3.07(0.76,12.37)	0.114	0.78(0.23,0.71)	0.699
CG/GG	CC	GG	GC/CC	1.30(0.35,4.93)	0.697	0.44(0.14,1.38)	0.16
CG/GG	CC	GA/AA	GG	0.72(0.20,2.54)	0.610	0.23(0.08,0.68)	0.008
CG/GG	CC	GA/AA	GC/CC	0.75(0.23,2.50)	0.644	0.30(0.11,0.84)	0.022
CG/GG	CT/TT	GG	GG	1.21(0.33,4.39)	0.772	0.32(0.10,1.01)	0.052
CG/GG	CT/TT	GG	GC/CC	0.73(0.21,2.48)	0.612	0.18(0.06,0.53)	0.002
CG/GG	CT/TT	GA/AA	GG	0.85(0.26,2.85)	0.797	0.28(0.10,0.81)	0.018
CG/GG	CT/TT	GA/AA	GC/CC	0.80(0.25,2.61)	0.712	0.36(0.13,0.97)	0.044
				P for interaction=0.084, interaction index=0.12		P for interaction=0.525, interaction index=2.10	

All tests were adjusted by age, sex and *H. pylori* infection. Abbreviation: GC, gastric cancer; GA, atrophic gastritis; CON, healthy controls.
